# Adrenergic Urticaria: An Updated Review

**DOI:** 10.7759/cureus.62171

**Published:** 2024-06-11

**Authors:** Kristin N Slater, Ahmad Abu-Zahra, Francisca Kartono

**Affiliations:** 1 Dermatology, Lincoln Memorial University DeBusk College of Osteopathic Medicine, Harrogate, USA; 2 Medicine, Michigan State University College of Human Medicine, East Lansing, USA; 3 Dermatology, MI Skin Center Dermatology Clinic, Northville, USA

**Keywords:** autoimmune disorders and urticaria, differential diagnosis of chronic urticaria, psychology and urticaria, urticarial eruptions, vasoconstriction in urticaria, stress-induced urticaria, adrenergic urticaria review, urticaria, adrenergic, adrenergic urticaria

## Abstract

It can be difficult to delineate the cause of urticarial eruptions, and in chronic cases, it can be a challenging condition to effectively treat. Several forms of urticarial eruptions are well documented and established. Our review focuses on a form of urticaria that is less commonly reported: adrenergic urticaria. In this review, we aim to consolidate the literature in the hopes that this urticarial subtype is considered in urticarial differentials, as well as highlight potential gaps in the research and future directions in treatment options.

## Introduction and background

Adrenergic urticaria (AU) is an urticarial eruption induced by stress and has been categorized as a physical form of urticarial eruption [1-8]. It is clinically defined by transient disseminated episodes of papules, wheals, macules, and plaques in severe cases that are characteristically surrounded by a white halo of vasoconstriction [1-6,8]. AU is described as a rare form of urticaria [1-2,4-5,7]. It was first described in 1985 by Shelly and Shelly as an “autonomic-system-dependent urticaria” with associated increases in plasma epinephrine and norepinephrine during the time of flare [8]. Given its relatively new categorization, several unknowns exist regarding AU’s pathophysiology, incidence, treatment, associations, and contributing factors. Additionally, with the rise in new biologic and immunomodulatory treatments for inflammatory dermatologic conditions, there may be additional options in the future for the management of AU. This review aims to cover the current literature on AU while identifying potential gaps in the literature and future directions for research to better understand AU and its associations and implications.

## Review

AU is considered to be a rare form of urticaria [1-2,4-5,7], although there is not enough information available in the literature to ensure it is a rare occurrence. AU may be misdiagnosed, potentially contributing to a percentage of idiopathic chronic urticarial eruption diagnoses. Additionally, a percentage may be misdiagnosed as another form of urticarial eruption, such as cholinergic urticaria, which shares some overlap in symptom profile [1]. In other cases, AU may be simply underreported and underdiagnosed [1]. We think that this potential gap in incidence may be related to its relatively new categorization as well as the limited research, educational resources, and awareness surrounding the diagnosis.

**Figure 1:**
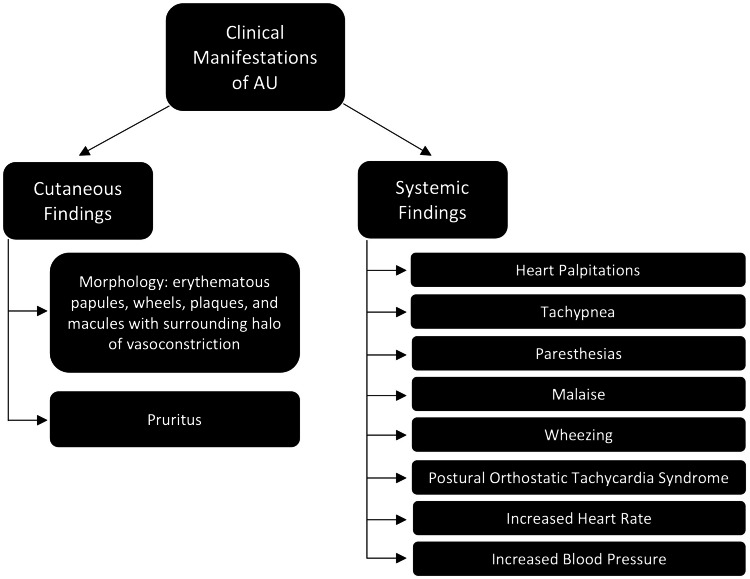
Clinical cutaneous and systemic manifestations of AU AU: adrenergic urticaria Image Credit: Kristin Slater, DO, MS

**Figure 2:**
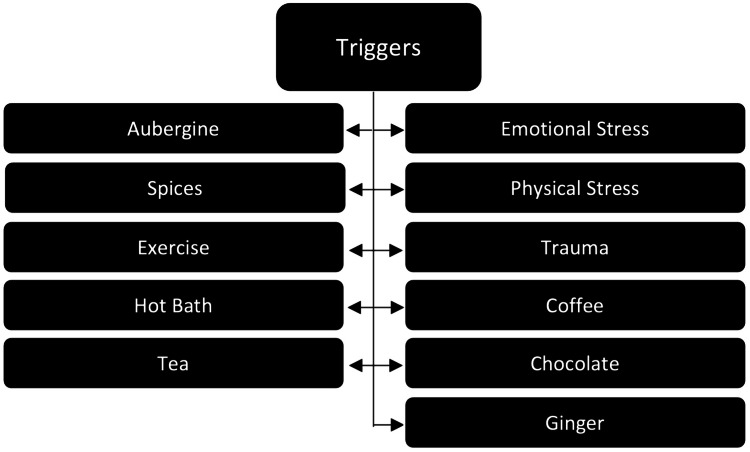
Documented triggers of AU AU: adrenergic urticaria Image Credit: Kristin Slater, DO, MS

The classic clinical cutaneous manifestations of AU are transient and disseminated and include its characteristic white halo of vasoconstriction surrounding erythematous papules, wheals, macules, and plaques in severe cases [1-6,8]. Pruritus can be present with cutaneous findings (Figure 1) [5,7,9]. Systemically, some manifestations have been associated with AU, including heart palpitations, tachypnea, paresthesias, malaise, wheezing, postural orthostatic tachycardia syndrome, increased heart rate, and increased blood pressure (Figure 1) [1,4,9]. These episodes are precipitated by numerous triggers, most notably emotional stress, but also include physical stress, trauma, coffee, chocolate, ginger, aubergine, spices, exercise, a hot bath, and tea (Figure 2) [1-8]. The episodes are transient, lasting anywhere from minutes to a half hour [3,5].

Although the pathogenesis of AU is unknown, a proposed mechanism was described by Hogan et al. in their 2014 review [1-2,5]. Their proposed mechanism suggested that a stressful event caused the activation of the brain stem’s locus coeruleus, releasing norepinephrine, which activates the amygdala via beta-1-adrenergic receptors [1]. This causes a downstream sympathetic response, which stimulates the adrenal medulla to release epinephrine and norepinephrine [1]. They hypothesize that it is the release of epinephrine by the adrenal medulla that causes the cutaneous vasoconstriction responsible for the characteristic white halo present in AU lesions [1]. Additionally, they hypothesize that the norepinephrine release by the adrenal medulla causes the cutaneous mast cell activation and degranulation, responsible for the erythema, vasodilation, and edema seen in the erythematous center of AU lesions [1]. This hypothesized mechanism also explains the reported increase in heart rate and blood pressure that is associated with AU flares, as well as the efficacy of propranolol in treating AU via non-selective inhibition of beta-adrenergic receptors [1].

**Figure 3:**
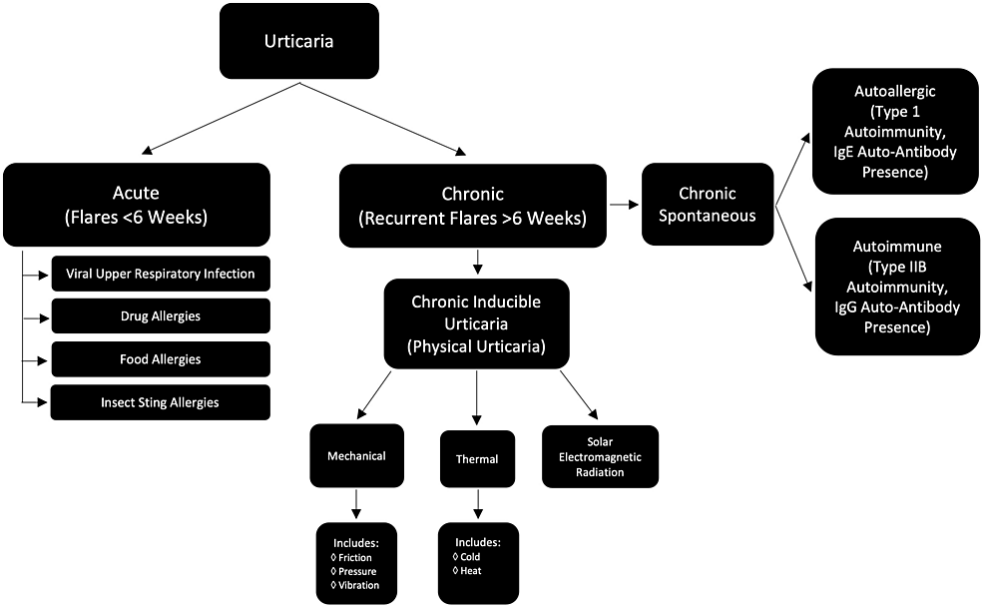
Acute and chronic groupings and subgroupings of urticaria Image Credit: Kristin Slater, DO, MS

**Figure 4:**
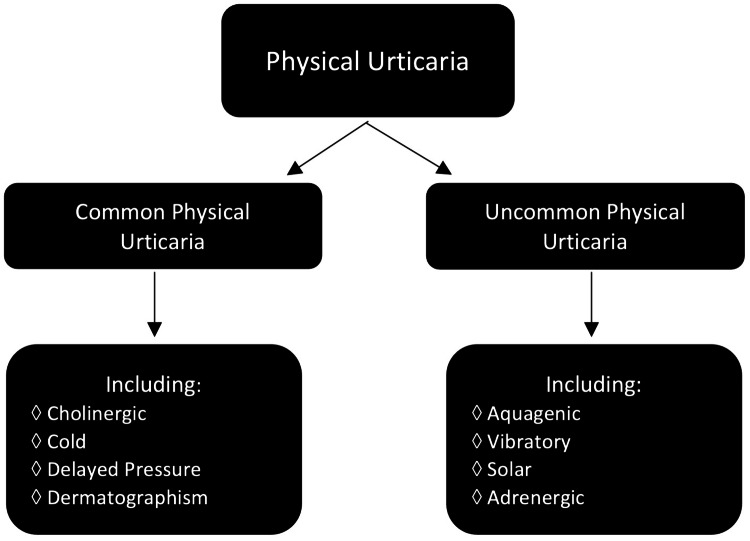
Broad alternative grouping: common and uncommon physical urticaria Image Credit: Kristin Slater, DO, MS

To delineate how AU is different from other forms of urticaria, it is important to understand the urticarial groupings. Broadly, urticarial eruptions can be acute (flares <6 weeks) or chronic (recurrent flares >6 weeks) [10]. Acute urticaria can be caused by many different variables, including viral upper respiratory infections, drug allergies, food allergies, and insect sting allergies [10]. For this review, chronic urticaria is the more relevant grouping, which includes the category of chronic spontaneous urticaria and its two subtypes: autoallergic (type 1 autoimmunity, IgE auto-antibody presence) and autoimmune (type IIb autoimmunity, IgG auto-antibody presence) [10]. Another known category is chronic inducible urticaria (physical urticaria), which includes subcategories based on its triggers: mechanical (which includes friction, pressure, and vibration urticaria), thermal (including cold and heat urticaria), and solar electromagnetic radiation (Figure 3) [1,10-11]. Physical urticarial groupings can be alternatively broadly subdivided into common physical urticaria (including cholinergic, cold, delayed pressure, and dermatographism) and uncommon physical urticaria (including aquagenic, vibratory, solar, and adrenergic) (Figure 4) [1]. These groupings have some overlapping symptoms, so it is helpful to keep a robust differential when diagnosing chronic urticarial eruptions.

Although the pathogenesis and pathophysiology of AU are largely unknown, there have been some reports documenting the association of AU with autoimmune disorders [1-2,5]. There is one case documenting AU in a patient with rheumatoid arthritis and melanoma, a case with AU in a patient with atopic skin reaction and thyroid autoantibody, a case of AU in a patient with anti-double-stranded DNA antibodies, and AU in a patient with vitiligo [2,5,12-13]. There are limited cases in the literature on AU, and consequently, there is limited information regarding AU’s associations. Filling this research gap could be beneficial in providing a more cohesive understanding of AU and its associations and implications.

Importantly, AU is fundamentally related to stress [1-8]. This makes understanding the psychological component important. It has been noted that some patients reported in the literature had a history of psychiatric diagnoses or a form of emotional lability [1,5]. Hyperactivity of the locus coeruleus is considered to play a role in hyperresponsiveness in posttraumatic stress disorder [14]. Stressful events activating the brain stem’s locus coeruleus and releasing norepinephrine are hypothesized to play a role in AU [1]. Further research investigating whether higher rates of PTSD or other psychiatric conditions altering the activity of the locus coeruleus are present in patients with AU could be advantageous in better understanding AU’s psychiatric associations. Research testing to see if there is general hyperactivity of the locus coeruleus in patients with AU could also be beneficial in better understanding AU.

Diagnosis and treatments for AU differ from those for other forms of urticaria. AU can be diagnosed by injecting 5 to 15 ng of epinephrine or 3 to 10 ng of norepinephrine in 0.02 mL of saline intradermally, resulting in the characteristic urticarial eruption [1-2,4-5]. Antihistamines used in AU have shown variable improvement [1-5]. There is a report documenting the successful treatment of AU with omalizumab [15]. There is also a report of refractory AU resistant to beta-blockers successfully treated with clotiazepam [16]. Additionally, there has been one reported case of dupilumab, an IL4 receptor antagonist, used successfully to treat AU [4]. The standard treatment for AU is propranolol 10-60 mg orally two to three times daily, depending on severity [1-5,7-8]. Allergic reactions to propranolol causing urticarial eruptions are rare but have been reported and are something to be aware of [17]. It is plausible that newer medications that have been used to treat other forms of urticarial eruptions could also be used in AU, given the successful use of omalizumab and dupilumab [4,15]. Other therapies could include older treatments such as IVIG, rituximab, TNFa inhibitors, and secukinumab [18]. Newer therapies currently under development that could potentially treat AU include Bruton's tyrosine kinase (BTK) inhibitors [18]. BTK inhibitors could be beneficial in treating multiple forms of urticarial eruptions, including AU, because of their effect on FcεRI-mediated mast cell activation, as well as B-cell functionality and the basophil signal cascade [18]. Because the mechanism of AU is not well understood, we do not fully know if newer alternatives would be effective. As the development of new, advanced immunomodulating drugs in dermatology continues, further research conducted to determine if these newer classes could be helpful in AU would be useful.

The long-term effects and general pathophysiology of AU are unknown. The limited available information on AU creates several literature gaps and corresponding research opportunities to fill these gaps. It is possible that AU is not as rare as it is reported to be and that misdiagnoses are occurring. Future research surrounding the association, correlations, mechanism of action, implications, and treatments would be advantageous for the further understanding of AU.

## Conclusions

AU is a form of urticarial eruption that is less commonly discussed. With awareness and education surrounding this form of urticarial eruption, we are hopeful that it will be more effectively diagnosed and managed in the future. More research is needed to delineate AU’s mechanism of action, potential treatments, and associations. The long-term effects and pathophysiology of AU are unknown, and limited literature is available. Future research to understand the role of AU’s psychiatric associations and corresponding mechanisms, as well as its potential association with autoimmunity, would be beneficial. Furthermore, as new immunomodulatory and targeted dermatologic therapies are used to treat other forms of urticarial eruptions, investigating AU’s pathophysiology and potential response to these therapies could be helpful in managing AU.
